# Investigating the Role of Food-Derived Peptides in Hyperuricemia: From Mechanisms of Action to Structural Effects

**DOI:** 10.3390/foods14010058

**Published:** 2024-12-28

**Authors:** Yu Han, Wanlu Liu, Kexin Li, Mingzhen Zhang, Xinqi Liu, Lu Li, Zhao Guo, He Li

**Affiliations:** 1 Key Laboratory of Geriatric Nutrition and Health, Beijing Technology and Business University, Ministry of Education, Beijing 100048, China; hancc0101@163.com (Y.H.); liuwanlu19990115@163.com (W.L.); 2230202116@st.btbu.edu.cn (K.L.); liuxinqi@btbu.edu.cn (X.L.); lil@btbu.edu.cn (L.L.); 2 Rushan Hualong Biotechnology Co., Ltd., Weihai 264500, China; 15069057959@163.com; 3 Department of Orthopaedics, The Affiliated Hospital of Hebei University, Baoding 071000, China

**Keywords:** bioactive peptide, hyperuricemia, constitutive relationship, mechanism of action

## Abstract

Hyperuricemia, a disorder of purine metabolism associated with cardiovascular disease, gout, and kidney disease, can be alleviated by food-derived peptides. However, the precise mechanisms remain unclear, hindering their development. This study reviews uric acid-lowering peptides from various sources, focusing on two pathways: inhibiting uric acid production and promoting excretion. Low-molecular-weight peptides (<1000 Da) exhibited superior uric acid-lowering effects. We further explored the relationships between amino acid composition and their target interactions. Peptides rich in cyclic amino acids (tryptophan, phenylalanine, and histidine) and containing small amounts of linear amino acids (leucine, cysteine, and glycine) demonstrated significant potential for lowering uric acid. These findings provide theoretical support for developing novel functional foods for the management of hyperuricemia.

## 1. Introduction

Hyperuricemia (HUA) is a metabolic disorder caused by the body’s excessive production or impaired excretion of uric acid (UA) and is usually due to purine metabolism disorders, renal insufficiency, or genetic factors [1,2]. In recent years, driven by the adverse effects of the modern lifestyle and diet, hyperuricemia has become an increasingly pervasive and heavy public health burden [3,4]. Chronic HUA results in the crystallization of UA in extremities and joints as well as in the kidneys, subsequently increasing the risks of gouty arthritis, cardiovascular disease, kidney disease, hypertension, hypothyroidism, obesity, and diabetes mellitus [5,6]. Thus, HUA is not only a metabolic disease but is also closely associated with a number of chronic diseases, making it a significant health risk factor; therefore, the prevention and management of the condition are crucial.

Currently, the main strategies for the treatment of HUA include pharmacologic therapy and dietary interventions. HUA is commonly treated with xanthine oxidase (XO) inhibitors (e.g., allopurinol, febuxostat, and topiroxostat), UA excretory agents or inhibitors of UA reabsorption (e.g., probenecid, lesinurad, and benzbromarone), and recombinant forms of UA oxidase (e.g., rasburicase and pegloticase), administered via enzyme therapy to metabolize serum UA into allantoin [7,8,9]. Although these drugs do provide some relief from hyperuricemia, their high cost and their potential to trigger various adverse effects in clinical use, including allergic reactions and toxicity to the kidneys and liver, are of significant concern [10]. Therefore, further research and development is required to improve low-molecular-weight drugs for the amelioration of HUA. In addition, numerous epidemiological studies have emphasized the benefits of a healthy diet and lifestyle in the mitigation and even prevention of this high-risk disorder [11].

Food-derived peptides are short-chain amino acids produced from food proteins through enzymatic digestion and microbial fermentation. These bioactive peptides have been found to exhibit a wide range of beneficial physiological effects, including antioxidant, hypotensive, antibacterial, antidiabetic, anticancer, and antiobesity properties [12,13,14,15,16,17]. Moreover, as highly active, stable, and specific biomolecules, food-derived peptides have garnered increasing attention for their potential to alleviate HUA, and several studies have demonstrated that these peptides can significantly lower UA levels in the body [18,19]. Thus, the administration of bioactive peptides, as dietary supplements or nutritional additives, has provided a novel approach to the management of chronic diseases.

Although several review articles have explored the mechanisms of UA-lowering peptides and their production strategies, the conformational relationships between the structural features of these peptides and their interactions with xanthine oxidase (XO) and related transporter proteins remain somewhat unclear [11,20,21]. Therefore, this review investigated and summarized the known protein hydrolysates and peptides with UA-lowering properties recently reported via various sources. We explored the action targets of food-derived UA-lowering peptides as well as the conformational relationships between these peptides and their targets. Specifically, this paper emphasizes two modes of action: the reduction in UA production through XO inhibition, and the promotion of UA excretion by modulating transporter proteins. The review also discusses the conformational relationships of peptides with their targets in terms of factors such as molecular weight, amino acid type, number, and position, and reveals their potential for UA reduction. These insights provide theoretical support and a reference basis for future research and the development of UA-lowering peptides in functional foods.

## 2. Pathogenesis and Inhibition of HUA

**Figure 1:**
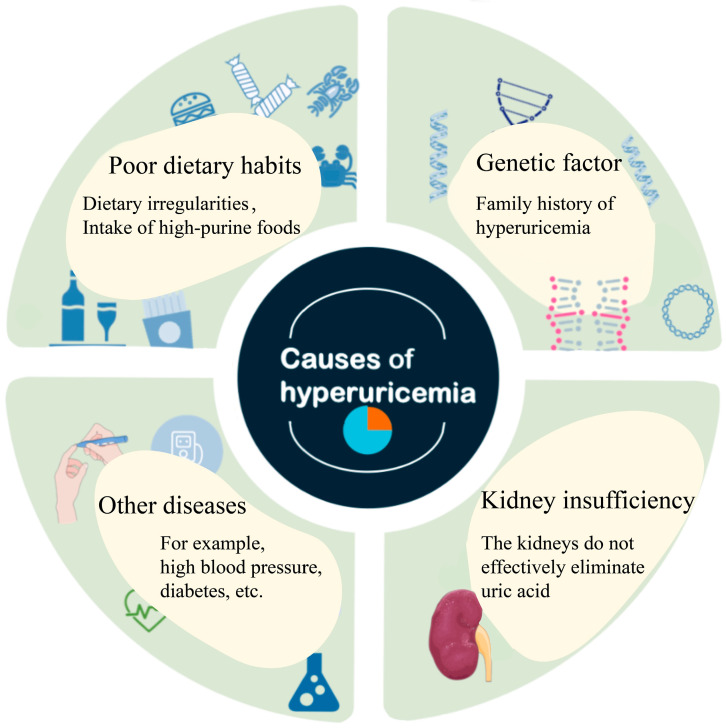
Causes of hyperuricemia.

The recent, rising global prevalence of HUA has triggered accompanying increases in not only gout but also the onset and progression of various associated systemic diseases, including renal disorders, metabolic syndrome, cardiovascular diseases, and cerebrovascular diseases. The accumulation of UA in the body occurs primarily through two mechanisms: the overproduction of UA (10% of cases) and insufficient excretion of UA (90% of cases) [22]. Factors such as an unhealthy diet and lifestyle, enzyme dysfunction in purine metabolism, renal disease, and certain pharmacological interventions can lead to excessive serum uric acid (SUA) levels, triggering HUA, as illustrated in Figure 1.

**Figure 2:**
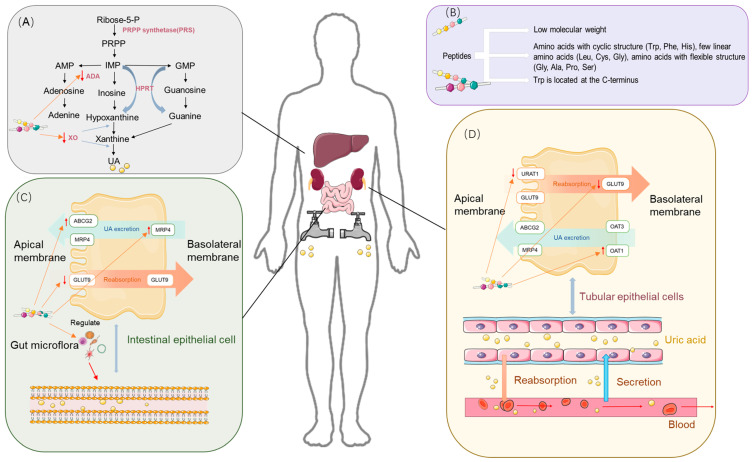
Uric acid-lowering mechanism of food-derived peptides. (**A**) Uric acid synthesis process and the role of peptides. (**B**) Structural features of characteristic bioactive food-derived peptides. (**C**) Uric acid excretion process in the intestine, transport proteins in intestinal epithelial cells, and the role of peptides. (**D**) Uric acid excretion process in the kidneys, transport proteins in renal epithelial cells, and the role of peptides.

Through an in-depth study of UA production and excretion processes, we identified that the two major physiological regulatory mechanisms by which UA levels are regulated include the inhibition of key enzymes involved in purine synthesis and the modulation of key transporter proteins in the kidneys and intestines to reduce UA reabsorption and promote its excretion [23] (Figure 2).

### 2.1. Inhibition of Uric Acid Synthesis

Approaches to the prevention and treatment of HUA aim to regulate SUA levels, in which key enzymes of purine metabolism play a critical regulatory role. Xanthine oxidase (XO) and adenosine deaminase (ADA) are key enzymes in the uric acid synthesis pathway. As shown in Figure 2A, the process of nucleic acid metabolism involves the enzymatic hydrolysis of nucleic acid to nucleosides, which are further hydrolyzed to free bases. The purine base is eventually converted to xanthine, which is subsequently oxidized to form UA by xanthine XO. XO, a low-specificity enzyme containing molybdenum (Mo), catalyzes the formation of xanthine from hypoxanthine, as well as the formation of uric acid from xanthine and the concomitant reaction to generate superoxide radicals. Uric acid became an end product as the body lost uricase during evolution [24]. ADA is a mercapto enzyme and catalyzes the generation of inosine (a precursor of hypoxanthine), which is ultimately oxidized by XO to form uric acid [25].

### 2.2. Improvement of Intestinal and Renal UA Excretion and Inhibition of UA Reabsorption

Usually, UA is transported from the blood to the urine for excretion through UA transporter proteins, which play roles in both reabsorption and secretion. The abnormal expression or function of these transporter proteins, impaired renal function, or the inhibition of transporter activity by medications or foods can result in decreased UA excretion, which increases the risk of HUA. Key UA reabsorption transporter proteins include urate transporter 1 (URAT1), glucose transporter 9 (GLUT9), and organic anion transporter 4 (OAT4). UA excretion transporter proteins include ATP-binding cassette sub-family G member 2 (ABCG2), organic anion transporter 1 (OAT1), multidrug resistance-associated protein 4 (MRP4), and Na+-dependent phosphate transporter (NPT).

Reabsorption of UA in the kidneys is primarily mediated by URAT1, GLUT9, and OAT4, as shown in Figure 2C. URAT1 is a reabsorption transporter protein expressed on the apical brush border membrane of proximal tubular epithelial cells in the kidneys [26], and SUA content is positively correlated with URAT1 expression [27,28]. GLUT9 is expressed in the basolateral membrane of the proximal renal tubular epithelium, and its main function is UA reabsorption, to which it contributes more than URAT1 [29]. OAT4 is expressed in the parietal membrane and mediates urinary organic anion reabsorption back into proximal tubular cells [30].

Urate excretion transport is mainly mediated by ABCG2, OAT3, MRP4, and OAT1 [31]. ABCG2, which is located in the apical membranes of proximal renal tubular epithelial cells and intestinal epithelial cells, plays a role in urate excretion in both the kidneys and the small intestine [32].

OAT3 and OAT1 are located in the basolateral membrane and are responsible for the initial stages of organic anion secretion and their transport from the blood across the basolateral membrane into proximal renal tubular cells [33]. MRP4, located predominantly in the apical membrane of proximal renal tubule cells, aids in the excretion of UA and other intracellular organic anions [34].

Similar to the kidneys, the gut plays an important role in the elimination of UA, which can be excreted through the interaction of transporter proteins with the intestinal flora, as shown in Figure 2C. The intestinal contribution to the elimination of UA has been documented to be approximately 30%, while the other 70% is eliminated through the kidneys [35]. ABCG2 is found in the proximal tubular epithelium and parietal membrane of intestinal epithelial cells and is involved in urate excretion in the kidneys and small intestine. Increased expression of ABCG2 was observed in the intestines of rats with impaired renal excretion [36], and increased mouse SUA was found in ABCG2-knockout mice. In addition [37], GLUT9 is located in the apical and basolateral intestinal epithelial cell membranes and is abundantly expressed in the jejunum and ileum, and intestinal epithelial cell-specific GLUT9 defects impair intestinal epithelial cell urate transport. One study found that GLUT9-deficient mice cause HUA with other complications. Dysregulation or dysfunction of these transport proteins leads to HUA and related metabolic diseases [38].

In addition, the gut microbiota indirectly or directly influences the excretion of UA [39]. Hyperuricemia (HUA) increases serum uric acid (UA) levels, altering the gut environment and leading to gut microbiota dysbiosis. This dysbiosis disrupts intestinal barrier integrity, increases gut permeability, and facilitates the translocation of bacteria and their metabolites into the bloodstream, triggering inflammatory responses. Moreover, the chronic inflammation associated with HUA elevates pro-inflammatory cytokine levels, further exacerbating microbial imbalances, thereby establishing a vicious cycle between inflammation and gut dysbiosis that accelerates the progression of HUA [38,39]. Thus, the gut microbiota is key to alleviating HUA and chronic inflammation in HUA. Whey protein peptide intervention in rats increased the number of thick-walled bacterial phyla and decreased some potential intestinal pathogens (*Bacteroides*), leading to the alleviation of HUA [40]. In addition, some prebiotics or probiotics can alleviate HUA through multi-targeted effects, e.g., Lactobacilli are able to reduce SUA by reducing the intestinal absorption of purines [41].

## 3. Food-Derived Peptides Alleviate HUA via XO Inhibition

A variety of food-derived peptides have been shown to exhibit XO inhibitory effects, as summarized in Table 1. For instance, peptides derived from the whey protein hydrolysates PEW and LLW were isolated, and their inhibitory effects on xanthine oxidase (XO) were investigated through in vitro experiments. The results showed that PEW and LLW significantly inhibited XO (IC_50_ = 3.46 ± 0.22 mM; IC_50_ = 3.02 ± 0.17 mM). The PEW peptides interacted with residues in the active cavity of XO through hydrogen and hydrophobic bonds [42]. Dong et al. reported that the soy-derived pentapeptides SHECN and SHCMN reduced the fluorescence signals of the amino acids Tyr and Phe in XO, as observed through fluorescence quenching analysis. This suggests that the peptides interact with the enzyme, possibly inhibiting its activity by affecting the environment around these amino acids [24]. Additionally, Lin et al. demonstrated that Ganoderma lucidum polysaccharide peptide (GLPP) reduced blood UA by 40.6% in HUA mice in a dose-dependent manner [43]. Peptides from other sources, including walnut protein (WDQWWW) [44], tuna (ACECD) [45], shark cartilage (YLDNY and SPPYWPY) [46], and yellowtail (WDDMEKIW) [47], have also been shown to have potent XO inhibitory activity.

Food-derived peptides with unique characteristics, such as molecular weight, amino acid sequence, and binding patterns, play an important role in inhibiting XO and ultimately lead to a reduction in UA synthesis. An expanded discussion is presented below.

### 3.1. Molecular Mass

**Figure 3:**
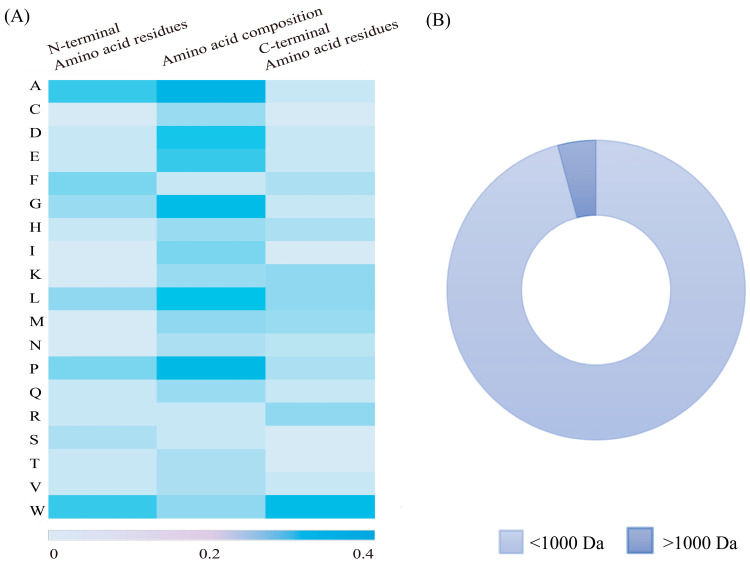
(**A**) Composition of amino acid residues in food-derived UA-lowering peptides and (**B**) percentage of low-molecular-weight peptides.

Food-derived peptides with a low molecular weight (<1000 Da) typically exhibit higher levels of UA-lowering activity than those with a high molecular weight (>1000 Da) [69]. This is primarily due to the increased difficulty with which high-molecular-weight peptides must enter XO active sites (Mo active site: 10 Å) [70] (Figure 2B). Our analysis of 48 sets of food-derived uric acid-lowering peptide sequences reported during the past five years revealed that peptides of <1000 Da accounted for approximately 95%, as graphically depicted in Figure 3B. In one study, the identification of hydrolysates obtained from ovigerous pomfret (*Trachinotus ovatus*) fish uncovered four fractions with varying molecular weight ranges. Among these, the low-molecular-weight peptides (<1000 Da) with XO inhibitory activity were significantly more prevalent than those with higher-molecular-weight (>1000 Da) fractions [51]. Similarly, peptides with high XO inhibitory activity in skipjack tuna hydrolysates were found to be predominantly within the 600 to 1000 Da range [45], while in rice hydrolysates, XO inhibitory peptides were mainly below 1000 Da and comprised 92.18% of the total [71]. In addition, the efficacy of XO inhibition is superior in shorter peptide chains, as demonstrated by the enhanced XO inhibitory activity exhibited after the optimization of long-chain peptides to those with shorter chains [61]. Compared with high-molecular-weight peptides, low-molecular-weight peptides (<1000 Da) offer several advantages: (1) they are more likely to penetrate cell membranes, enter cells, and interact with enzymes and transport proteins, and (2) their higher bioavailability facilitates more effective absorption and utilization by the body, and their therapeutic effects are exerted more rapidly to alleviate HUA.

### 3.2. Amino Acids with Bicyclic Structure

Amino acids with cyclic structures demonstrate significant XO inhibition effects, as illustrated in Figure 3A. Previous studies have shown that peptides with cyclic structures access the optimal binding site of XO more readily compared with linear peptide molecules [72].

Tryptophan has a unique bicyclic (indole ring) structure that contributes significantly to XO inhibition [48,51] (Figure 2B). Its structure is similar to that of XO inhibitors (e.g., allopurinol), and the indole ring structure is similar to the C6 and C5 ring structures of allopurinol. The indole ring of tryptophan consists of a benzene ring and a pyrrole ring, endowing it with a permanent dipole moment that allows pointing from the nitrogen atom (N1) of the indole ring to the carbon atom (C5). This dipole moment is capable of forming hydrogen bonds or ionic interactions with polar residues of the XO enzyme, thus facilitating the binding of tryptophan to the enzyme and influencing the substrate recognition and catalytic activity of the enzyme [24]. In particular, this unique indole system not only enhances cation–π interactions but may also trigger anion–π interactions [67]. Research indicates that peptides containing Trp residues (e.g., FPAW, LLPW, and WLLP) exhibit markedly higher and more effective inhibitory activity than those without (e.g., FHLP) [51]. Nongonierma et al. found that among 20 amino acids and 15 dipeptides with XO-inhibiting properties, 6 polypeptides (W, RW, WV, VW, KW, and IW) contained Trp residues [48], and Li et al. demonstrated that WDQWWW, with four Trp residues, had a 63.16% lower IC_50_ value compared with WDQWW, which has only two Trp residues [44]. And Trp was found to interact with amino acid residues, including Leu873, Glu802, Ser876, Phe914, Arg880, Phe1009, Val1011, Thr1010, Ala1078, Leu1014, and Ala1079, in the same way as allopurinol. The position of tryptophan residues also influences XO-inhibiting activity. As shown in Figure 3A, Trp is found significantly more frequently at the C-terminus compared with other amino acids. Hou et al. reported that LLPW and WLLP, which share the same amino acid composition but differ in their Trp residue positioning, exhibit different levels of XO inhibition, with the Trp residue at the C-terminus exhibiting approximately 10 times the XO-inhibiting activity of WLLP [51]. The same conclusion was reached by Li et al. [63], who found that the XO-inhibiting activity of PPKNW was higher than that of WPPKN, and Nongonierma et al. [19] observed similar results in comparisons between VW and WV.

### 3.3. Amino Acids with Monocyclic Structure

Monocyclic amino acids, such as phenylalanine (Phe) and histidine (His), are also known to contribute to lowering UA. Phe, an aromatic amino acid with a benzene ring, has demonstrated significant inhibitory effects on XO (Figure 2B). Hou et al. identified Phe and leucine (Leu) as potent amino acids in reducing UA production [51]. Zhao et al. reported that Phe within the peptides extracted from oyster protein hydrolysate (GGYGIF) engaged in π–π stacking with XO [48]. The optimization to WGWGW, which enhances multiple π–π stacking interactions, is a key factor in improving Vina scores, and Phe is crucial for binding efficiency. He et al. found that Phe in FW contributed to a more stable XO–FW complex through π–π interactions with Phe-914 of XO, resulting in a higher degree of binding efficiency [54]. Moreover, it is suggested that Phe can significantly enhance the inhibitory effect of Trp-containing peptides. Tyrosine (Tyr), which also features a monocyclic structure, is limited in its ability to effectively block XO interactions by its polar -OH group, which keeps it away from crucial hydrophobic channels [54].

His, which is a heterocyclic amino acid containing an imidazole group, contrasts with Phe in that its cyclic structure enables both π–π and π–cation interactions with XO [54] (Figure 2B). He et al. observed that FH binds similarly to quercetin, with π–π stacking between the His side chain in FH and Phe-914 in the XO active cavity being a key interaction [54]. Further research has revealed that the His side chain in EKIWHH, extracted from fish, carries a basic charge that potentially facilitates binding with residues in the XO active site [50].

### 3.4. Amino Acids with Linear Structures

Linear amino acids also play a crucial role in XO regulation, as illustrated in Figure 2B. A strong correlation has been demonstrated between the XO-inhibiting activity of peptides and hydrogen atom donor amino acids, such as cysteine (Cys). The tuna-derived UA-lowering peptide ACECD (IC_50_ = 7.23 mM), which contains two Cys residues, was found to exhibit significant XO inhibitory activity in comparison with that of PGACSN and WML [45], probably due to the S-H bond in the sulfhydryl group of Cys, which readily loses hydrogen atoms and forms stable hydrogen bonds. Additionally, Liu et al. demonstrated both in vivo and ex vivo that AAAAGAKA inhibited XO activity, with glycine forming hydrogen bonds with Asn19 on XO [62]. These findings all provide valuable insights for the further design and development of peptide drugs with enhanced XO inhibitory activity.

## 4. Food-Derived Peptides Alleviate HUA by Modulating UA Transporter Proteins

Numerous food-derived peptides have demonstrated the potential to modulate UA levels by affecting transporter proteins. Moringa peptides, for example, were found to effectively inhibit the expression of URAT1 and promote the expression of ABCG2 in the kidneys of potassium oxybate-induced hyperuricemic rats, thereby enhancing renal urate excretion and alleviating HUA [73]. Similarly, tilapia hydrolysate has been observed to reduce the mRNA expression of URAT1. These findings indicate that food-derived peptides can influence UA metabolism and excretion via the regulation of transporter proteins. Studies that focus specifically on the regulation of UA-related transporter proteins by peptides are limited; however, peptides have been shown to modulate transporter proteins in other contexts, such as antiglycation [74], antimicrobial [75], and anti-inflammatory [76] processes, which suggests their potential to modulate UA transporter proteins.

Approximately 70% of HUA cases are attributed to renal excretion disorders, in which around 90% of UA is reabsorbed by the kidneys [77]. Reabsorption transporter proteins (e.g., GLUT9 and URAT1) are particularly crucial in UA regulation, as they are primarily responsible for its transport [78]. Beyond the kidneys, the intestine also plays a significant role in UA elimination [79], with ABCG2, which is highly expressed in intestinal tissues, contributing to one-third of UA excretion. Despite these insights, the exact structures and regulatory mechanisms of these transporter proteins remain unclear. Thus, this review will explore the functions and mechanisms of these three key transporter proteins and examine how peptides can facilitate their UA-lowering effects through pharmacological inhibition.

### 4.1. mRNA Modulation

Another way in which peptide molecules may influence UE excretion is by modulating the expression of mRNAs for related transporter proteins, thereby altering the expression of these proteins. Currently, drugs in clinical trials or under development are focused primarily on either XO or URAT1 inhibition; however, peptide molecules have the potential to simultaneously and synergistically inhibit (or promote) multiple transporter proteins, such as URAT1, GLUT9, and ABCG2, through multi-targeted effects. It has been found that oligopeptide-rich sea cucumber hydrolysate was able to down-regulate the mRNA expression of URAT1 and up-regulate the mRNA expression of OAT1 and ABCG2 [43]. Although the simultaneous inhibition of URAT1 and GLUT9 and promotion of ABCG2 mRNA expression can reduce UA levels, the effects of different inhibitors on transporter protein mRNA expression can vary [80,81,82]. Co-incubation studies of mouse renal tubular epithelial cells with inhibitors revealed that transporter protein mRNA expression was not significantly affected by inhibitor treatment [78].

### 4.2. Occupation of Transporter Protein Sites

#### 4.2.1. Roles of Spatial Positional Resistance and Flexible Structures

**Figure 4:**
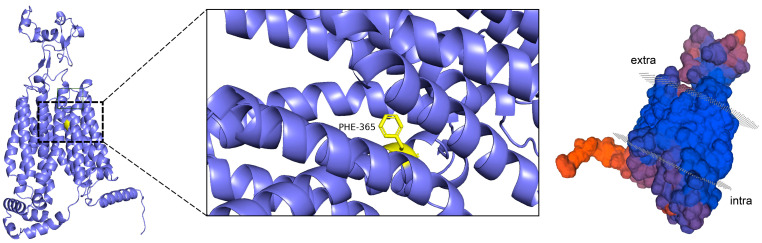
Visualization of URAT1 homology modeling.

Peptide molecules can hinder the binding of UA to transporter proteins by occupying key binding sites through spatial site blocking, and their flexible structures facilitate access to these binding cavities. Spatial site blocking is, therefore, an effective strategy for URAT1 inhibition, achieved either by interfering with the binding of UA to URAT1 or by inducing conformational changes in URAT1. A variety of small-molecule inhibitors have been developed to modulate the functions of URAT1, GLUT9, and ABCG2 by binding to the core of these transport proteins and spatially obstructing the transport of UA through the substrate channel. Verinurad, for instance, specifically inhibits hURAT1 by occupying key residues in the substrate channel, namely, Cys-32, Ser-35, Phe-365, Met-25, and Ile-481, and effectively reducing URAT1 transport [78]. Similarly, Tan et al. found that this inhibitor exerts its effects by targeting three binding sites (Ser-35, Phe-365, and Ile-481) in hURAT1 [83]. Phe-365 is particularly critical in the active pocket of transporter proteins [83,84], as can be seen in Figure 4.

This key binding site, located within the URAT1 channel, significantly enhances the probability of inhibitor binding to reactants, the mechanism of which is crucial for the design of effective inhibitors. The Phe-365 residue in hURAT1 (equivalent to Tyr-365 in rURAT1) reportedly binds tightly to the inhibitor through hydrophobic π–π stacking, thereby significantly affecting its affinity. The Ile-481 mutation has been shown to have no significant effect on URAT1 activity, whereas mutations at Ser-35 and Phe-365 were found to significantly reduce the inhibitory effect of the inhibitor CDER167 [78]. Flexible linkers, which are short sequences connecting proteins, peptides, or other biomolecular fragments, play a significant role in the actions of small-molecule inhibitors. For example, the insertion of a methylene unit between the triazole and naphthyridine in lesinurad enhanced its URAT1 inhibitory activity [83]. Additionally, the linker between the aromatic moieties of an inhibitor is known to be active during biological activity [85], and it has been shown that the addition of a flexible linker enables the dual-target inhibition of URAT1 and GLUT9 and ensures the maintenance of minimal inhibitory activity against other targets [78].

#### 4.2.2. Regulation of Transport Proteins Through Spatial Site Resistance and Flexible Amino Acids

Food-derived peptides that can form π–π stacks with key binding sites, like Phe-365, demonstrate more potent UA-lowering effects, similar to the peptides described above that inhibit XO (Figure 2B). Peptides with cyclic structures are more likely to form effective stacking interactions and, therefore, offer significant potential for the inhibition of both XO- and UA-related transporter proteins. Additionally, the introduction of flexible structures into such peptides can further enhance their ability to bind to transporter proteins. Flexible amino acids, characterized by a high degree of rotational freedom and simpler side chains, provide the highest degree of structural flexibility [86]. Thus, incorporating flexible amino acids (e.g., Gly, Ala, Pro, and Ser) or linkers into the peptide chain may improve binding affinity and inhibitory efficacy. For instance, the tripeptides RPK (IC_50_ = 1.867 mM) and FLR (IC_50_ = 1.148 mM) both exhibit significant UA-lowering activity; the lower IC_50_ value of the latter is due to its flexible amino acids. These mechanisms highlight the potential of peptide molecules as UA-lowering therapeutics and warrant further investigation to validate their roles and optimize their designs.

## 5. Conclusions and Prospects

This paper summarized the mechanisms of UA-lowering action, focusing on two pathways: the inhibition of UA production and the promotion of UA excretion, as well as their relationship with relevant targets. Based on the results of this review, we hypothesize that peptides with specific structural features, particularly those containing cyclic amino acids, such as Trp, Phe, and His, are effective inhibitors of XO. Among these, Trp at the C-terminal position shows superior UA-lowering effects, with its inhibition correlating positively with Trp content in peptides. In addition to cyclic amino acids, structural amino acids such as Leu and Cys also play essential roles.

Future studies should focus on the following aspects of this disorder: (1) Peptide preparation methods should be optimized. Targeted enzymatic hydrolysis techniques for peptides rich in Trp or His should be developed. This can be achieved by employing enzymes with specific cleavage sites designed to selectively hydrolyze peptide bonds adjacent to Trp or His residues, thereby maximizing the yield of bioactive peptides with desired structural characteristics and enhanced UA-lowering efficacy. (2) Mechanistic studies on UA transporter proteins should be conducted. The role of peptides in regulating UA transport and their structural interactions with transporter proteins should be investigated. (3) Future studies should prioritize clinical trials to verify the safety and efficacy of UA-lowering peptides in humans, addressing differences in UA metabolism, such as the presence of uricase in rodents. Additionally, research should focus on identifying key binding sites of peptides for XO and UA transporter proteins in humans, providing a basis for their therapeutic application in managing gout and related diseases. (4) Stability and bioavailability should be investigated. Future studies should evaluate the stability and bioavailability of food-derived UA-lowering peptides, focusing on practical applications. The Caco-2 intestinal epithelial cell model can be used to simulate gastrointestinal digestion and assess peptide absorption. These studies will help identify peptides with optimal stability and bioavailability for therapeutic use.

## Figures and Tables

**Table 1:** Structural characterization, type of inhibition, and inhibitory activity of UA-lowering peptides from various sources. ↓ indicates a decrease in activity due to the inhibitory effect of food-derived peptides.

No.	Source		Peptide Sequence	Key Amino Acids	Inhibition Type	Experimental Model	Molecular Weight (Da)	Dose–Response Relationship	Ref.
1	Fish and marine life	Oyster	WGWGW	W	XOD↓	In vitro	690.75	IC_50_ = 1.86 ± 0.024 mM	[48]
2		Pacific white shrimp	AEAQMWR	W	XOD↓ GLUT9↓ URAT1↓	In vitro	891.01	IC_50_ = 8.85 ± 0.05 mM	[49]
3		Small yellow croaker	WDDMEKIW	W	XOD↓	In vitro	1122.24	IC_50_ = 3.16 ± 0.03 mM	[47]
4		*Scophthalmus maximus*	WDDMEKIWHH	W, H	XOD↓	In vitro	1259.38	IC_50_ = 1.598 mM	[50]
5		*Katsuwonus pelamis*	ACECD	-	XOD↓	In vitro	539.58	IC_50_ = 7.23 mM	[45]
6		*Trachinotus ovatus*	LLPW	W, P	XOD↓	In vitro	527.31	IC_50_ = 4.17 ± 0.12 mM	[51]
			WLLP	W, P			527.31	IC_50_ = 43.06 ± 0.73 mM	
			FPAW	W, P			519.25	IC_50_ = 3.81 ± 0.18 mM	
			FHLP	PH			512.27	IC_50_ ≥ 50.00 mM	
7		*Shark cartilage*	YLDNY	-	XOD↓		686.71	5 mg/kg body weight intravenously and 50 mg/kg body weight orally had a significant XOI	[46]
			SPPYWPY	W, P		Male Wistar rats	908.99		
8		Bonito	WML	W	XOD↓	Male Sprague–Dawley rats	448.21	-	[52]
9		Sardines	FLR	-	XOD↓	HK-2 cells	435.28	IC_50_ = 1.148 mM	[53]
10		Tuna	RPK	P	XOD↓	Sprague–Dawley rats	400.21	IC_50_ = 1.867 mM	[54]
			FH	H	XOD↓		302.33	IC_50_ = 25.70 mM	
11		*Auxis thazard*	PDL	P	XOD↓	In vitro	344.87	IC_50_ = 12.73 ± 0.32 mM	[55]
			SVGGAL	-			504.26	IC_50_ = 11.12 ± 0.18 mM	
12		Antarctic Krill	DIFDPL	-	XOD↓	Male Balb/c mice	718.79	-	[56]
13		Pacific cod bone–flesh mixture	FF	-	XOD↓		312.36	IC_50_ = 0.80 mM	[57]
			YF	-			328.36	IC_50_ = 0.52 mM	
			WPW	W, P		In vitro	487.55	IC_50_ = 1.68 mM	
			WPDARG	W, P			700.74	IC_50_ = 0.40 mM	
			YNVTGW	W			738.78	IC_50_ = 0.23 mM	
14	Milk	Whey protein	ALPM	P	XOD↓	Male Sprague–Dawley rats	430.22	IC_50_ = 7.23 ± 0.22 mM	[58]
			LWM	W			448.21	IC_50_ = 5.01 ± 0.31 mM	
15		Whey protein	PEW	W, P	XOD↓	Caco-2 cells	430.45	IC_50_ = 3.46 ± 0.22 mM	[42]
			LLW	W			430.54	IC_50_ = 3.02 ± 0.17 mM	
16		Whey protein	GL	-	XOD↓		188.20	IC_50_ = 10.20 ± 0.89 mM	[18]
			PM	P		In vitro	246.30	IC_50_ = 23.82 ± 0.94 mM	
			AL	-			202.20	IC_50_ = 34.49 ± 0.89 mM	
			AM	-			220.20	IC_50_ = 40.45 ± 0.92 mM	
17	Plant	Rice	AAAAMAGPK-NH2	P	XOD↓ URAT1↓	Kunming and nude mice	785.97	XOI = 23.4 ± 1.4 U/L (1 mg/kgAAAAMAGPK-NH_2_)	[59]
18		Soy	SHECN	H	XOD↓	LO2 cells	588.59	The UA concentration of 2.5 mg/mL SHECN was 88.98 ± 0.78 μg/mL	[60]
19		Shelled *Oryza sativa* fruit	AAAAGA	-	XOD↓ URAT1↓ GLUT9↓	Kunming and nude mice	430.45	100nMAAAAGA UA concentration of 35.32 ± 0.001 mg/L	[61]
20		Shelled *Oryza sativa* fruit	AAAAGAKAR	-	XOD↓	Sprague−Dawley male rats	785.91	-	[62]
21		Walnut	WDQW	W	XOD↓	In vitro	633.65	-	[44]
22		Walnut	WPPKN	W, P	XOD↓		640.8	IC_50_ = 26.25 ± 0.177 mM	[63]
			ADIYTE	-		Male Sprague–Dawley rats	710.7	IC_50_= 23.73 ± 0.29 mM	
23		Kidney bean	DWYDIK	W	XOD↓	In vitro	838.9	XOI = 68.63 ± 5.07% (1 mg/mLDWYDIK)	[64]
24		Macadamia nut	PGPR	P	XOD↓	In vitro	425.49	IC_50_ = 24.84 ± 0.02 mM	[65]
			GPY	P			335.36	IC_50_ = 30.44 ± 0.33 mM	
			HGGR	H			425.45	IC_50_ = 24.89 ± 0.19 mM	
25	Fungi	*Pleurotus ostreatus*	FCH	H	XOD↓	Male Sprague–Dawley rats	405.47	IC_50_ = 2.04 mM	[66]
26	Others	Hemoglobin	LIGLW	W	XOD↓	In vitro	600.75	IC_50_ = 1.09 ± 0.03 mM	[67]
27		Egg protein	EEK	-	XOD↓	In vitro	404.41	IC_50_ = 0.334 mM	[68]

## Data Availability

No new data were created or analyzed in this study. Data sharing is not applicable to this article.
